# Impact of Nanoclays Addition on Chickpea (*Cicer arietinum* L.) Flour Film Properties

**DOI:** 10.3390/foods13010075

**Published:** 2023-12-25

**Authors:** Ángel Cobos, Olga Díaz

**Affiliations:** Área de Tecnología de Alimentos, Departamento de Química Analítica, Nutrición y Bromatología, Facultad de Ciencias, Universidade de Santiago de Compostela, 27002 Lugo, Spain; angel.cobos@usc.es

**Keywords:** biopolymer films, bentonite, halloysite, Cloisite 20A, mechanical properties, microstructure

## Abstract

Chickpea flour is an affordable natural blend of starch, proteins, and lipids, which can create films with suitable properties as an eco-friendly packaging material. Nanoclays’ incorporation into natural biopolymers enhances the barrier properties of the resulting nanocomposites, so they could improve the properties of flour films. The objective of this work was to assess the influence of three types of nanoclays (halloysite, bentonite, and Cloisite 20A) at two concentrations on the characteristics of chickpea flour films. In general terms, when the lowest dose (5%) was added, no or very slight significant differences with the control were observed in most parameters, except for thermal stability and opacity, which increased, and solubility, which decreased. At the highest concentration (10%), films containing any of the nanoclays demonstrated greater thermal stability, opacity, and rigidity while being less soluble than those without nanofillers. Bentonite exhibited superior film structure distribution compared to other nanoclays. At the highest concentration, it had the most significant impact on modifying the properties of chickpea flour films, increasing their tensile and puncture strengths while decreasing elasticity and water vapor permeability. The incorporation of nanoclays into chickpea flour films could be a useful technique to enhance their properties.

## 1. Introduction

Vegetal flours are interesting raw materials for the preparation of biopolymer composite films due to their low cost, high availability, and biodegradability, which can contribute to finding substitutes for petroleum-based materials for food packaging. In the last few years, many studies related to the properties of flour-based films of various origins have been published [1,2]. Among them, chickpea flour has recently received attention because it is a natural mixture of starch, proteins, and lipids able to produce films of appropriate characteristics that are adaptable to food protection. The effects of pH, plasticizer (glycerol) concentration, and the addition of an antioxidant (gallic acid) on film properties have been investigated [3,4]. There has been research into the preservation capability of sunflower oil and seeds in chickpea flour films [5,6]. In addition, films based on chickpea flour and chitosan or polyethylene oxide added to curcumin [7,8] have been studied.

The reinforcement of natural biopolymer films with nanofillers has gained strong interest due to the improvement of the barrier properties of the nanocomposites compared to those of the original biopolymer film. Nanofillers include nanoclays, organic, and inorganic, among others [2]. Attention has been focused on nanoclays because of their high availability, low cost, and significant improvements in biopolymer film properties [9,10]. Among clay fillers, montmorillonite (MMT) is the most widely studied. It is a hydrated alumina-silicate-layered clay composed of one aluminum hydroxide octahedral sheet between two silica tetrahedral sheets. When used in nanocomposites, these silicates consist of two-dimensional layers that are several microns long and whose thickness is on the nanometric scale [9]. Bentonite is the most representative of this type of clay and consists of 90% MMT; it is an untreated hydrophilic sodium-montmorillonite nanoclay that originates from the weathering processes of volcanic glass [11]. The hydrophilic surface of natural montmorillonites was a barrier to their use in petroleum-based polymers. For this reason, organo-montmorillonites, products of the chemical modification of the hydrophilic surface with organic cations promoting an organophilic surface, were developed. There are a variety of commercial organically modified clays for food packaging applications. Among them, Cloisite 20A is an organically modified nanoclay that contains the modifier agent dimethyl, dihydrogenated tallow, and quaternary ammonium [12]. Halloysite is another type of nanoclay, a natural aluminosilicate clay of the kaolin group, with a hollow nanotubular structure that varies in length from the submicron scale to even >30 μm and an internal diameter of 10–100 nm. This structure makes it useful for biologically active molecule encapsulation [2,13]. Halloysite structure is stable in solutions at pH values of 2 to 11; at alkaline pH (>8) it is well dispersed, and, particularly at pH 11, the degree of dispersion reaches a maximum [13]. The addition of montmorillonite and halloysite nanoclays to starch films improves their barrier and mechanical properties [10,14]. Bentonite and kaolin are specified as generally recognized as safe (GRAS) for food contact in the United States and have been approved as additives in bulk form in the European regulations [15].

MMT nanoparticles have been added to some vegetal flour (amaranth, banana, and rice) films, improving their mechanical properties and decreasing water vapor permeability [16,17,18]. The literature about nanoparticle addition to chickpea flour films is very scarce; only one article describes the effect of the addition of nanocellulose particles on the properties of these films [19]. However, studies about the effects of the addition of nanoclays on the properties of chickpea flour films have not been carried out.

The aim of this work was to evaluate the impact of three nanoclays (halloysite, bentonite, and Cloisite 20A) at two concentrations on the water vapor permeability, solubility, swelling, antioxidant capacity, color, opacity, mechanical properties, microstructure, thermal stability, and biodegradability of chickpea flour films.

## 2. Materials and Methods

### 2.1. Materials

Commercial chickpea flour (*Cicer arietinum* L.) (Legumbres Pedro S.L., Alcalá de los Gazules, Spain) (Hijo de Macario Marcos S.L., Salamanca, Spain) was purchased from a local market. According to the manufacturer, the composition of the product was 22.3% protein, 4.9% fat, 58.2% carbohydrates, 8.1% moisture, and 2.5% ash. Halloysite and bentonite (Nanomer PGV) nanoclays were supplied by Sigma-Aldrich (St. Louis, MO, USA) and Cloisite 20A by BYK-Chemie GmbH (Wesel, Germany).

### 2.2. Films Preparation

Nanoclays (halloysite, bentonite, and Cloisite 20A) were dispersed in distilled water at two concentrations: 5% and 10% (*w*/*w*) on a chickpea flour basis. The dispersions were stirred at 1000 rpm in a Arex Digital PRO stirrer (VELP Scientifica Srl., Usmate, Italy) for 5 min at room temperature (20 °C) and were incubated at 60 °C for 1 h in a water bath with continuous shaking (mod. Unitronic OR; J.P. Selecta, Barcelona, Spain); this method has been used to delaminate nanoclays of different hydrophobicity [20]. After, they were sonicated in an ultrasonic bath (40 kHz; mod. Sonorex Digiplus DL 512 H; Bandelin Electronic GmbH, Berlin, Germany) for 45 min [14] at 60 °C. The dispersions were cooled down to room temperature and used to prepare the film-forming solutions. Chickpea flour (6 g/100 mL of water) was added and mixed by low stirring in a magnetic stirrer for 30 min at 20 °C. Then, glycerol (Panreac, Barcelona, Spain) was added in a proportion of 1.8% (*w*/*v* of water; 30% *w*/*w* on chickpea flour basis), and the dispersions were stirred for an additional 15 min. Then, the pH was adjusted to 10.0 with 2 N NaOH. The dispersions underwent heat treatment at 80 °C for 20 min in a digital magnetic stirred (VELP Scientifica Srl., Usmate, Italy) connected to a temperature controller (VTF Digital Thermoregulator, VELP Scientifica Srl., Usmate, Italy). Film-forming solutions (0.22 g cm^−2^) were poured into Plexiglas Petri dishes of 14 cm diameter and dried at 35 °C for 20 h in an air force cabinet. After, the dried films were kept at 25 °C and 45% relative humidity for 48 h prior to peeling them off in a climate chamber (Ensayos Terlab S.L., Barcelona, Spain) and were stored in desiccators at room temperature and 45% relative humidity for further testing. Control films were also obtained without adding nanoclays. The experiments were performed in triplicate.

### 2.3. Film Thickness

Film thickness was determined using a 0–25 mm electronic digital micrometer with 0.001 mm resolution (Selecta, Barcelona, Spain) at 20 points of three films selected randomly.

### 2.4. Water Vapor Permeability

Water vapor permeability (WVP) was measured according to the method described by Díaz et al. [3], based on the ASTM E-96-93 method [21], with some modifications. Film samples (0.032 ± 0.001 m exposed area diameter) were attached to glass cups containing dried silica gel, placed in desiccators with a saturated solution of potassium carbonate, and maintained in a chamber at 25 °C. The weight gain of the cups was measured twice a day for 5 days. Measurements were performed in triplicate. WVP was calculated using the following formula:

WVP (ng s^−1^ m^−1^ Pa^−1^) = (*w* × *L*)/(*A* × Δ*t* × Δ*P*) (1)

where *w* is the weight gain of the cell (ng) during the time of permeation Δ*t* (s); *L* is the film thickness (m); *A* is the permeation area (m^2^); and Δ*P* is the partial water vapor pressure difference (Pa) across the two sides of the film.

### 2.5. Dry Matter Content, Solubility, Swelling Property, and Density

Dry matter content, solubility, and swelling degree were determined according to the method described by Yildiz et al. [7]. Film samples (2 cm × 2 cm) were weighed (*W*_1_) and dried at 105 °C until a constant weight (*W*_2_). The dried samples were then immersed in 25 mL of water at 25 °C for 24 h. Afterwards, any excess water was carefully removed from the surface by using filter paper, and the sample was weighed again (*W*_3_). Finally, the sample was dried at 105 °C for 24 h, and the final weight was determined (*W*_4_). Dry matter, solubility, and swelling degree were calculated as:

(2) $$Dry\;matter\left(\%\right)=100-\left[\frac{W_{1}-W_{2}}{W_{1}}\times 100\right]$$

(3) $$Swelling\;degree\left(\%\right)=\left.\frac{W_{3}-W_{2}}{W_{2}}\times 100\right.$$

(4) $$Solubility\left(\%\right)=\left.\frac{W_{2}-W_{4}}{W_{2}}\times 100\right.$$

The density of films was determined in accordance with the method of Sun et al. [22]. The density was determined by dividing the weight of the film by its volume. The volume of the film was calculated by multiplying its area by its thickness. All measurements were conducted in triplicate.

### 2.6. Color and Opacity

Color parameters and opacity of films were determined in the CIE L*a*b* color space according to the methods reported by Díaz et al. [23], using a spectrophotometer X-Rite (mod. SP60; Grand Rapids, MI, USA). The color of three different films in three random positions was measured.

The difference in color (Δ*E*) of films with regard to the control film color values was calculated as:

(5) $$∆E=\sqrt{{\left(L^{*}-L_{0}\right)}^{2}+{\left(a^{*}-a_{0}\right)}^{2}+{\left(b^{*}-b_{0}\right)}^{2}}$$

where L*, a* and b* are the color values for the film added to nanoclay and *L*_0_, *a*_0_ and *b*_0_ are the color values for the control film.

The yellowness (*YI*) and whiteness (*WI*) indexes of films were calculated according to Saberi et al. [24] using the equations:

(6) $$YI=\frac{142.86b}{L}$$

(7) $$WI=100-\sqrt{{\left(100-L\right)}^{2}+a^{2}+b^{2}}$$

where *L*, *a,* and *b* are the color parameter values of the sample.

Opacity values were calculated according to Márquez-Reyes et al. [25] as:

(8) $$Opacity\left(\%\right)=\left.\frac{Y_{B}}{Y_{W}}\times 100\right.$$

where *Y_B_* is the opacity of the film against a black background and *Y_W_* is the opacity of the film against a white background.

### 2.7. Antioxidant Activity

The DPPH radical-scavenging capacity of the films was determined using the method described by Vargas et al. [26]. Film samples (1 cm^2^) were submerged in tubes containing 3.9 mL of a 0.06 mM methanolic DPPH (2,2-diphenyl-1-picrylhydrazyl; Sigma-Aldrich, St. Louis, MO, USA) solution. The tubes were maintained in darkness at 25 °C for 2 h and 30 min. The film samples were separated, and the absorbance of the solution was measured at 517 nm in a spectrophotometer (mod. 6850 UV/Vis; Jenway, Bibby Scientific, Stone, UK). Methanol was used as a blank. A calibration curve was prepared with DPPH [4], using different concentrations of DPPH in methanol (0.006–0.06 mM). The determinations were carried out in duplicate. Antioxidant activity was expressed as milligrams of DPPH degraded per gram of dry film weight and was calculated as follows:

Antioxidant activity (mg DPPH/g dry film) = *C* × dilution rate/dry film weight in g (9)

where *C* is the value obtained from the calibration curve as milligrams of DPPH per mL of the difference between the absorbances of the DPPH reagent and the sample.

### 2.8. Mechanical Properties

Tensile strength, elongation at break, elastic modulus, puncture strength, and puncture deformation were tested according to the ASTM D882 method [27], as described by Díaz et al. [23]. After cutting, film samples were stored in a desiccator at 20 °C and 45% RH for 48 h before analysis. A texturometer mod. EZ Test and the Trapezium2 Data Processing System software version 1.03SP (Shimadzu Corporation, Tokyo, Japan) were used for mechanical property determination.

### 2.9. Fourier Transform Infrared Spectroscopy (FTIR)

The attenuated total reflectance-Fourier transform infrared spectra of films were obtained with a Jasco FT/IR-4600 equipped with the ATR Pro One accessory with an internal reflectance element of ZnSe (wave number range of 15,000–550 cm^−1^) (Jasco Corporation, Tokyo, Japan). Determinations were conducted in triplicate. The spectra were measured between 400 and 4000 cm^−1^ by co-adding 16 scans at 1 cm^−1^ resolution. Data treatments were performed with PeakFit software version 4.12 (SYSTAT Software, Richmond, CA, USA), according to Díaz et al. [3].

### 2.10. Thermogravimetric Analysis (TGA)

Thermogravimetric analysis of films was conducted using a thermogravimetric analyzer with differential scanning calorimetric capability (Mettler Toledo, mod. TGA/DSC1; Schwerzenbach, Switzerland), in accordance with the method described by Díaz et al. [3]. The PeakFit software (version 4.12; SYSTAT Software, Richmond, CA, USA) was used to calculate the first derivative of the TGA curve (DTG).

### 2.11. Scanning Electron Microscopy (SEM) Analysis

The microstructure of the film samples was observed by scanning electron microscopy using a JEOL-JSM 6360LV scanning electron microscope (Jeol, Ltd., Tokyo, Japan) operated at 15 kV. The working distance was 10 mm. Sample preparation was carried out as described by Díaz et al. [23]. Surface and cross-sectional images were taken at 200× and 1000× magnification, respectively. The roughness of film surfaces was measured from SEM images using ImageJ software version 1.54h (U.S. National Institutes of Health, Bethesda, MD, USA) [28].

### 2.12. Biodegradation Test

The biodegradability of films was conducted using the method described by Piñeros-Hernandez et al. [29]. Film samples (2 cm × 2 cm) were placed on a plastic mesh and buried at 1.5 cm depth in plastic trays containing organic soil. They were then incubated at 20 °C for 7 days. Then, the samples were recovered and photographed.

### 2.13. Statistical Analysis

Data evaluation were carried out using IBM SPSS Statistics for Windows version 28.0.1.0 (IBM Corporation, Armonk, NY, USA). Prior to the statistical analysis of the results, the data were tested for outliers and for normal distribution using the Kolmogorov-Smirnov test. One-way ANOVA and the least significant difference test were used to test and compare, respectively, the statistical significance of differences among means. For all mean evaluations, a significance level of *p* < 0.05 was used.

## 3. Results and Discussion

### 3.1. Water Vapor Permeability, Thickness, Dry Matter Content, Solubility, Swelling, and Density

Water vapor permeability, thickness, dry matter content, solubility, swelling, and density values of films are shown in Table 1.

Water vapor permeability significantly decreased in the films with bentonite at both concentrations compared with control films, and it was lower than that of the other films with nanofillers when added at 10%. Halloysite and Cloisite 20A additions did not significantly affect this parameter. Bentonite has been reported as more effective in decreasing WVP than other nanoclays, including MMT and organically modified MMT in soy protein films [30]. Films prepared with amaranth flour and bentonite also had a significantly lower WVP [16]. Unmodified MMT decreased this parameter in rice flour films as well [18]. This effect has been attributed to the interaction of the hydrophilic polymers (starch, proteins) with the clay, making the hydrophilic sites less available for water molecules [16]. Hydrogen bonding interactions between the hydroxyl groups of the polymer and the nanoparticles could result in a denser polymer matrix, which would reduce WVP [17]. The decrease of WVP has also been attributed to the formation of a tortuous path by the clay sheets dispersed in the polymer matrix when intercalation occurs; this makes difficult the diffusion of water vapor molecules throughout the material [16]. Although tortuosity effects exerted by halloysite addition to starch films have been mentioned in the literature, they do not seem to be enough to reduce the water permeability of films, probably due to the water affinity and the morphology (nanotubes) of this nanoclay [31].

Cloisite 20A films showed the highest thickness values for both concentrations, which was significantly different from control films. No significant differences among the other composite and control films were found. Frangopoulos et al. [32] also observed an increase in the thickness of chickpea starch films added to organically modified MMT, which they attributed to the large particle size of the nanoclay and to the increase in film solid content. However, bentonite was the nanoclay with the largest particle size (up to 25 μm), and it did not have any effect on thickness; Cloisite 20A particle size was lower than 10 μm. In addition, none of the nanofillers affected the dry matter content of the films (Table 1). The thickness increase could be due to the interaction of this organically modified MMT with chickpea flour compounds (mainly starch but also with protein and lipids) and to changes in film structure. Cloisite 20A structure is more “non-polar” than bentonite, enabling a greater interaction with hydrophobic compounds [33]; this could have happened with the lipids contained in chickpea flour, increasing thickness.

The dry matter content of films was not significantly affected by nanoclay addition. A similar result was reported by Orsuwan and Sothornvit [17] in banana flour nanocomposite films.

Nanoclays incorporation significantly decreased the solubility of chickpea flour films in relation to control ones. Halloysite and Cloisite 20A films at a concentration of 10% showed lower values than films with 5% nanoclay, while this parameter was not affected by the concentration in bentonite films. Water solubility also decreased in banana flour and potato starch composite films, probably due to the blockage of water diffusion into the structure by the nanoclay, stronger interactions between the molecules through hydroxyl groups and hydrogen bonds, and a more dense and crystalline structure of the films [14,17,34,35].

Cloisite 20A addition to films significantly decreased swelling compared to control films; halloysite incorporated at 10% also reduced swelling values. Cloisite 20A and halloysite nanocomposites results are consistent with the water solubility reduction reported by other authors [14]. This effect has been attributed to the interactions between biopolymer and nanoclay through hydrogen bonds; thus, free water molecules would exhibit weaker interactions with nanocomposite films compared to biopolymer films without nanoclays [14]. However, bentonite addition significantly increased film swelling and was higher in films with 5% nanoclay. Increases in swelling capacity of bentonite-added starch films have been reported by other authors [36,37]. The negatively charged surface of bentonite may attract water molecules, especially at nanoclay concentrations. Although a decrease in swelling at high bentonite concentrations has been observed, this property improves when the films make contact with NaOH solutions, and this effect is intensified when the bentonite amount increases. Bentonite interlayer magnesium ions are affected by sodium content. High sodium content significantly alters the natural swelling and cation exchange capacity of bentonite by affecting the interlamellar magnesium ions present in this nanoclay. Smectites such as bentonite have a high swelling capacity when sodium is the interlamellar cation. It has been reported that the swelling capacity of bentonite can be increased by up to seven times in an alkaline solution [36,37]. As a result, complete dissociation of individual crystals can occur, leading to a high degree of dispersion; this effect is enhanced by ultrasound treatment applied to the film [36]. During the preparation of chickpea flour composite films, pH was adjusted to 10.0 by NaOH solution addition, and ultrasounds were used to ensure complete homogenization of nanoclay suspensions. These treatments could affect the swelling properties of bentonite-added films.

Halloysite-added films showed significantly higher densities than control, and values were not affected by the concentration. Bentonite only increased this property at the highest concentration. Cloisite 20A significantly decreased density when added at 5%. Differences in nanoclay densities might influence the density of films; according to the manufacturers, the values for nanoclays are: 1.80 g/cm^3^ for Cloisite 20A, 2.6 g/cm^3^ for bentonite, and 2.53 g/cm^3^ for halloysite. However, the differences could also be attributed to changes in the interactions among polymeric matrix molecules due to the presence of nanoclays. In the case of halloysite and 10% bentonite films, the nanoclays could induce tighter binding with the matrix, and the distance between molecules could have been shortened, producing more compact structures with thicknesses similar to those of the control films. In the case of Cloisite 20A films, weaker interactions could occur, which significantly reduced the density values and increased thickness. Density modifications due to changes in polymer-molecule interactions have been reported in chitosan films when polyphenols were added [22].

### 3.2. Color

CIE L*a*b* color values, total color difference (Δ*E*), yellowness index, whiteness index, and opacity results are shown in Table 2.

L* values were influenced by both the concentration and type of nanoclay. Films with 10% nanoclays showed significantly lower values of L* than those with 5% nanofillers. Compared to control films, 5% halloysite films were lighter, while films with 10% bentonite and 10% Cloisite 20A were darker. No significant differences among films with these two nanoclays were observed. Other authors reported higher lightness in films with unmodified MMT compared to Cloisite 20A [38]. However, the dispersion procedure of the nanoclays and the nature of the polymer matrix (agar) were different, so these factors could influence the results.

Halloysite addition did not modify a*; 10% Cloisite 20A and both bentonite films showed significantly higher values of this parameter (they were less green than the other films). In bentonite films, the increase in a* values could be due to the brownish color exhibited by the nanoclay.

Regarding the b* value, the addition of nanoclays at a concentration of 10% produced films more yellow than at 5%, which is in accordance with the increase in b* when increasing MMT concentration observed by other authors [32,39]. In comparison with control films, significant differences were only found in 5% halloysite films and in 10% Cloisite 20A films that displayed lower and higher b* values, respectively.

The total color difference *(*Δ*E*) showed significant changes with the addition of nanoclays compared to control films. In all cases, Δ*E* values were higher than 1, which means that color differences can be noticed by a standard observer [40]. Only experienced observers may detect differences for both bentonite films and 5% Cloisite 20A films that reached values between 1 and 2, while inexperienced observers could perceive differences for halloysite films and 10% Cloisite 20A films (values between 2 and 3.5).

The films with the highest ΔE values showed significant differences in yellowness and whiteness indexes with control films. In comparison with them, 5% halloysite films were less yellow and whiter, and 10% Cloisite 20A films were yellower and less white. No significant differences among the indexes of the other films were found.

Nanoclays incorporation into chickpea flour films increased their opacity, and the values were higher at the highest concentration. The decrease in transparency has been attributed to the blocking effect of nanoparticles, which is proportional to the size and nanoparticle concentration in films [41,42]. Light transmission would be blocked by particles larger than the visible wavelength, reducing the transparency of films. Hong et al. [43] suggested that this effect could be caused by an incomplete intercalation of nanoclays in the polymer matrix, which might be exfoliated or dispersed as stacked particles of large size enough to affect this property in polyethylene/nanoclay/starch composite films.

### 3.3. Mechanical Properties

Table 3 shows the mechanical property values of films.

Halloysite addition significantly increased elongation at break at the two concentrations and also improved tensile strength. In general, it is reported that nanoclay addition involves decreasing elongation at break [42,44]; however, the improvement of this property in starch-based biodegradable biopolymer films due to the addition of halloysite has been described, although it depends on the concentration added, with a maximum at 10% nanoclay [45]. Elongation at break value increases has been observed in agar/chitosan and regenerate cellulose films, which has been attributed to the good dispersion of halloysite inside the film matrix and the interactions among film components [46,47]. Elastic modulus, puncture strength, and puncture deformation were not affected by this nanoclay.

Bentonite was the nanoclay that produced films with the highest tensile strength, elastic modulus, and puncture strength values at any concentration. At 10%, it significantly decreased elongation at break, which indicates that concentration is a determinant factor in this property. The concentration of bentonite affects the mechanical properties of starch films [37]. Cloisite 20A, at a concentration of 10%, significantly increased tensile strength and elastic modulus and decreased elongation at break. In addition, it reduced the puncture strength of films. Both bentonite and Cloisite 20A additions decreased puncture deformation.

Tensile strength and elastic modulus improvements have been associated with the increase in the number of hydrogen bonds between the film matrix and nanoclay particles, which provided greater resistance to forces applied to the films and reduced the mobility of polymer molecules. These effects may be related to the degree of intercalation/exfoliation of the nanoclays as a consequence of their compatibility and dispersion in the matrix [12,42,44]. The differences in mechanical properties between bentonite and Cloisite 20A could be attributed to the higher affinity of the former with the film matrix due to its hydrophilic nature, which makes it more compatible with starch and proteins in chickpea flour.

### 3.4. Antioxidant Activity

Table 4 shows the DPPH radical scavenging capacity of films.

The antioxidant capacity of films was not improved by nanoclays addition except with 5% bentonite; even halloysite decreased the antioxidant capacity of films. Decreases in the DPPH radical scavenging activity of chitosan films when kaolinite was introduced have been reported [48]. Bentonite effects were affected by the concentration added, increasing the values at 5% and decreasing them at 10% in relation to control films. Chickpeas possess antioxidant properties attributed to the presence of polyphenolic compounds and peptides and the protein-phenolic compound complexation; however, the type of chickpea used for flour manufacturing in Europe contains lower amounts of phenolic compounds [3]. Kim and Oh [49] studied the interactions between halloysite nanotubes and hydrophilic MMT with albumin, and they observed that protein-nanoclay interactions (probably of electrostatic nature among charged groups of both protein and nanoclays) seem to occur on the surface of nanoclay particles. MMT showed the strongest interaction with protein and induced higher denaturation of albumin than halloysite. This effect on protein structure could influence the antioxidant activity of chickpea proteins as well as other film characteristics, such as mechanical properties. An excessive denaturation or an increase in interactions of proteins with bentonite at the highest concentration might decrease the antioxidant capacity of films.

### 3.5. Fourier Transform Infrared Spectroscopy

**Figure 1:**
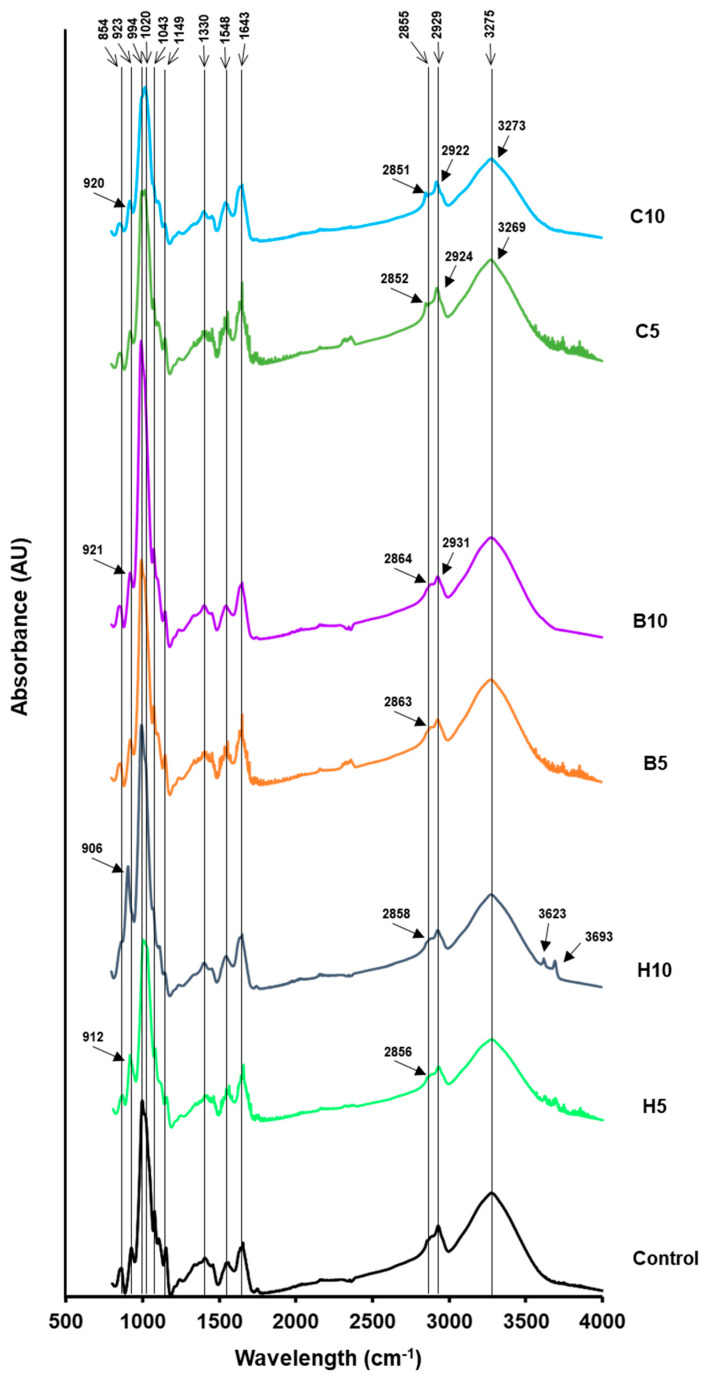
FTIR spectra of chickpea flour films added to nanoclays. Control, control film; H5, films with 5% halloysite; H10, films with 10% halloysite; B5, films with 5% bentonite; B10, films with 10% bentonite; C5, films with 5% Cloisite 20A; C10, films with 10% Cloisite 20A.

Figure 1 shows the FTIR spectra of chickpea flour films added to nanoclays.

Typical bands, associated with proteins and carbohydrates (800–1800 cm^−1^ and 2800–3700 cm^−1^), were observed. The band at 854 cm^−1^ has been related to C–C skeletal vibrations, and the band at 923 cm^−1^ to the glycosidic bonds of starch [3]. In this band, a wave number decrease was observed mainly when halloysite was added (906–912 cm^−1^) but also in films with 10% bentonite and Cloisite. These results might be due to an increased interaction of nanoclays, especially at high concentrations, with the glycosidic bonds of starch.

Peaks between 900 and 1050 cm^−1^ have also been assigned to the clay bonds of Si–O–Si and Si–O, and their intensity could increase upon intercalation of montmorillonites into the matrix [50,51,52]. However, no changes in bands at 950–1100 cm^−1^ have also been reported in soybean polysaccharide films with sodium montmorillonite [39]. In chickpea films, bands at 906 cm^−1^ in 10% halloysite films and at 994 cm^−1^ in 10% halloysite and bentonite films showed higher intensities than control films, while the intensity of these bands decreased in Cloisite-added films. These results might be related to the higher intercalation of the hydrophilic nanoclays compared to the more hydrophobic Cloisite 20A in the matrix.

Three bands have been associated with the structure order of starch: 993 cm^−1^ to the crystalline structure of starch, the intramolecular hydrogen bonding of hydroxyl groups, and the water sensitivity; 1043 cm^−1^ to the number of ordered regions of starch; and 1020 cm^−1^ to the amorphous region [53].

Two ratios, derived from the absorbance of these three bands, are frequently computed to estimate the short-range ordered structure of starch. The 993/1020 cm^−1^ ratio corresponds to the way in which the double helices are organized within crystals and to the sensitivity to hydration, while the 1043/1020 cm^−1^ ratio signifies the degree of organization in highly crystalline regions; both ratios are higher in more ordered starch structures [54,55]. Table 4 shows the values of these ratios in chickpea flour films. The addition of a high amount of halloysite and bentonite to films significantly increased the 993/1020 cm^−1^ ratio compared to control films, which has been related to double helices reaching a more ordered structure in the short order range inside crystallites [54,56]. Films with 10% Cloisite 20A showed significantly lower values, which could be attributed to a more disorganized structure of starch. The addition of low amounts of nanoclays did not significantly affect this ratio.

Nanoclays addition decreased 1043/1020 cm^−1^ ratio values in all samples, although the significant lowest values were found for 5% halloysite and 5% bentonite-added films. In general, and according to this ratio, nanoclays interactions with the film matrix decreased the order in more crystalline regions of starch, reflecting a weaker aggregation between helices [57].

Bands at 1075, 1104, and 1149 cm^−1^ have also been attributed to the stretching of starch bonds [3]. Peaks assigned to Amide I, II, and III modes (1643, 1548, and 1330 cm^−1^, respectively) were also observed [3,58,59]. The band at 1643 cm^−1^ had also been assigned to water absorbed in the amorphous regions of starch [60].

Peaks detected at 2855 and 2929 cm^−1^ were attributed to the symmetric and asymmetric stretching vibrations of C–H bonds (–CH_2_ groups) present in the polysaccharide structure [39]. The wavelengths of these bands shifted to lower values in Cloisite 20A films (2851–2852 and 2922–2924 cm^−1^) which may involve interactions between this nanoclay and the starch structure. Conversely, these bands shifted to higher values in halloysite and bentonite films, which suggests a decrease in their interactions with –CH_2_ groups of starch.

The absorbance and area of peak at 1149 cm^−1^, assigned to the stretching of the C–O–H glycosidic bonds of starch, decreased in nanoclay-added films compared to control films. This reduction has been attributed to strong interactions of –OH with oxygen-bound carbons [61].

The wide absorption band observed at 3275 cm^−1^ was associated with the stretching of the free, inter- and intramolecularly bonded –OH groups between nearby molecules [62]. In Cloisite 20A-added films, the 3275 cm^−1^ band shifted to lower wavenumbers (3269–3273 cm^−1^). It has been reported that this shift indicates an increase in the formation of intermolecular hydrogen bonds between the hydroxyl groups of the nanoclay and the matrix [63,64]. Organic MMT causes the disruption of inter- and intramolecular hydrogen bonds present among starch granules, exposing hydroxyl groups. This, in turn, results in the formation of new hydrogen bonds between starch chains and nanoclay layers [65]. When the concentration of Cloisite was higher, the wave number of this band rose compared to that of 5% addition; the number of interactions might decrease, probably due to the increase in aggregate formation among nanoclay molecules. The addition of the other two nanofillers to films did not provoke this effect, suggesting a decrease in interactions and weaker bonding. However, broadening of this band in bentonite films was observed; this change has also been attributed to the formation of new hydrogen bonds in MMT-starch films [66]. 

Bands at 3623 cm^−1^ and 3693 cm^−1^, that were only observed in halloysite added films, have been related to the O–H stretching of the inner-surface hydroxyl groups and inner hydroxyl groups, respectively, of the spectrum of this nanoclay [62].

### 3.6. Thermogravimetric Analysis

**Figure 2:**
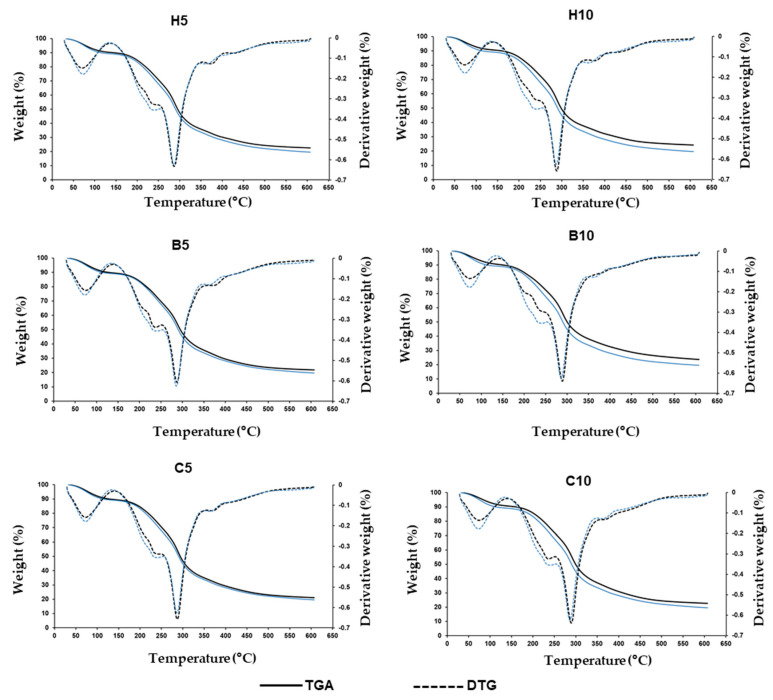
Thermogravimetric analysis (TGA) and derivative thermogravimetric (DTG) curves of chickpea flour films added to nanoclays. H5, films with 5% halloysite; H10, films with 10% halloysite; B5, films with 5% bentonite; B10, films with 10% bentonite; C5, films with 5% Cloisite 20A; C10, films with 10% Cloisite 20A. Blue lines are control films; black lines are films added with nanoclays.

Thermogravimetric analysis (TGA) and the first derivative of the TGA results (DTG) curves of the thermal degradation pattern of nanoclay-added films are shown in Figure 2.

All graphs exhibited a similar pattern, demonstrating three stages of weight loss; this suggests that the presence of nanoclays did not modify the thermogravimetric behavior of films. The first weight loss phase, which occurred between 32 and 140 °C, was associated with the desorption of free water and water linked to the matrix by hydrogen bonding [3]. A temperature peak at 75–78 °C was detected in all samples. Weight loss values varied from 9.32 to 10.88%.

In the second stage, which happened in the range of 140–250 °C, the glycerol-rich phase and the structurally bound water in the film were evaporated, and low-molecular-weight protein-carbohydrate compounds were degraded. The films showed a temperature peak between 239 and 243 °C, and weight losses were between 17.94 and 21.27%.

The third stage (between 250 and 600 °C) was related to the degradation of protein and carbohydrate components of the film’s backbone. Significant differences among samples in the temperature peak corresponding to this phase were observed (Table 4). Nanoclay-added films showed significantly higher peak values than control, exhibiting the highest value with 10% Cloisite 20A, followed by 10% bentonite films. Significant differences in the weight losses corresponding to the three phases among the film samples were not observed.

The remaining mass at the end of heating and after the removal of volatile compounds consisted of a carbon residue. The residual weights of nanoclay-added films were significantly higher than those of the control and were proportional to the amount of nanoclay added (Table 4). Significant delays in weight loss at high temperatures when MMT content increases have been reported [67]. The greatest values were found in films with 10% halloysite and 10% bentonite, while they were significantly lower in 10% Cloisite 20A films. The reduction of residual weight in organically modified MMT compared to unmodified MMT has been attributed to the thermal decomposition of the organic modifier [67,68]. The rise in thermal stability, observed in the increase of the final residue and the temperature at which the maximum decomposition rate happened (Table 4), has been attributed to several reasons: the thermal stability of nanoclays, the impediments in starch chain segmental mobility by the addition of nanoclays, and the formation of a winding path in the matrix structure, obstructing the flow of liquids and gases and the heat flux [34,69,70].

### 3.7. Film Microstructure

**Figure 3:**
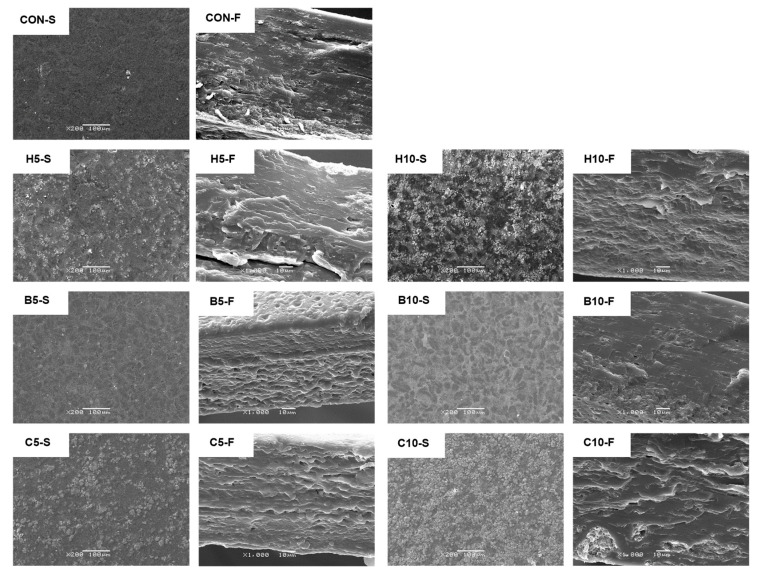
The microstructure of chickpea flour films is enhanced with nanoclays. CON-S and CON-F control film surface and fracture, respectively; H5-S and H5-F, surface and fracture of film with 5% halloysite, respectively; H10-S and H10-F, surface and fracture of film with 10% halloysite, respectively; B5-S and B5-F, surface and fracture of film with 5% bentonite, respectively; B10-S and B10-F, surface and fracture of film with 10% bentonite, respectively; C5-S and C5-F, surface and fracture of film with 5% Cloisite 20A, respectively; C10-S and C10-F, surface and fracture of film with 10% Cloisite 20A, respectively.

The SEM micrographs of the surface and a cross-section of chickpea flour films added to nanoclays are displayed in Figure 3. The roughness profiles obtained by SEM surface image analysis are shown in Figure S1. The control films showed a homogeneous surface and a compact and dense structure with a small number of aggregates. The addition of nanoclays increased the coarseness and roughness of the surface, although its intensity varied with the type and content of nanoclay. This effect of nanoclay concentration was also observed by other authors in the surface and fracture images of films [39,42,67]. Bentonite films exhibited smoother surfaces than those of the other films added to nanoclays (Figure S1). The highest degree of agglomeration and roughness was observed in halloysite and Cloisite 20A films, especially at 10% addition. The rougher fracture surface observed when 10% Cloisite 20A films were added, compared to 5% films, has been attributed to the formation of intercalated and disordered intercalation arrangements at high levels of MMT [67]. Halloysite films exhibited cracks and voids, and Cloisite 20A showed clearly visible large aggregates, which suggests poor interaction with the matrix. The white strands observed in MMT-added films could be attributed to nanoclay platelets [67].

Bentonite films showed aggregates of smaller size and more homogenously distributed than those of the other nanoclays. This lower roughness and the more compact structure of films with bentonite could be attributed to better interactions between the negatively charged bentonite and the positively charged starch [37] in comparison with halloysite and Cloisite 20A, which showed lower affinity with the film matrix. The presence of a more compact and homogeneous structure when hydrophilic MMT is added has also been observed in agar films [38]. The dense and compact structure of 10% bentonite films could be related to their higher tensile and puncture strengths and lower water vapor permeability (Table 1 and Table 3).

### 3.8. Biodegradability

**Figure 4:**
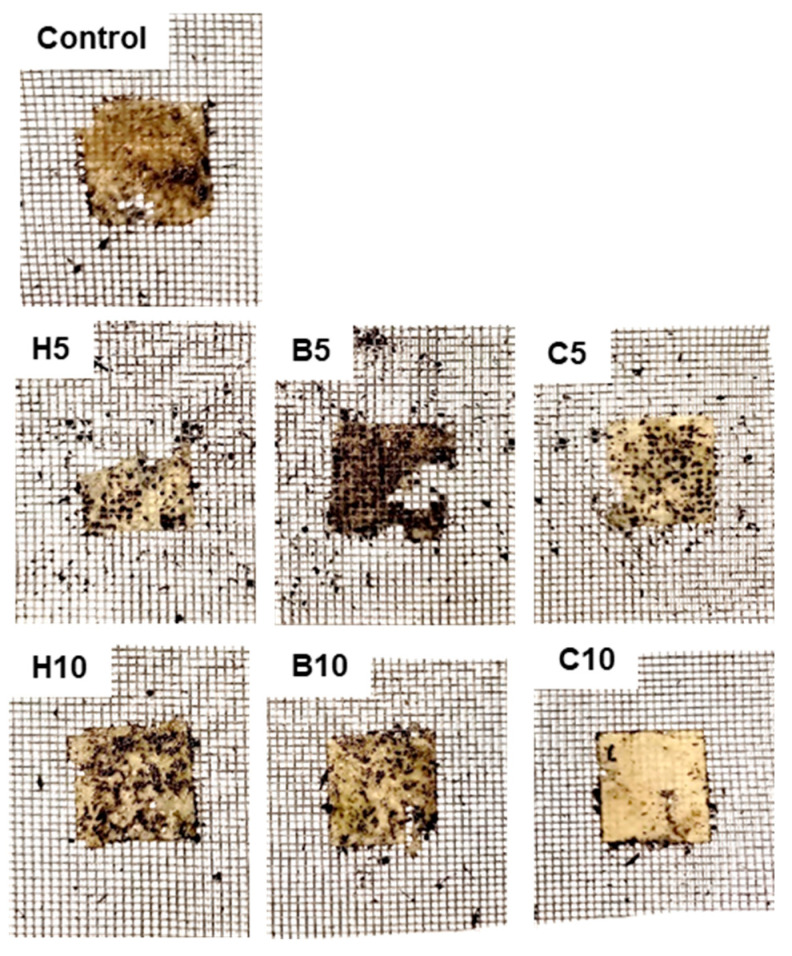
Biodegradability of chickpea flour films added to nanoclays. Control, control film; H5, film with 5% halloysite; H10, film with 10% halloysite; B5, film with 5% bentonite; B10, film with 10% bentonite; C5, film with 5% Cloisite 20A; C10, film with 10% Cloisite 20A.

Figure 4 shows the biodegradation test results of the nanoclay-added films. Degradation was higher in films with a lower amount of nanoclays, mainly in those added with halloysite and bentonite, in which some fragments disappeared. Their degradation was higher than that of control films, while at 10% nanoclay addition, the appearance of these films was similar to control. Cloisite 20A films showed higher stability, especially at 10% nanoclay addition. It has been reported that MMT modified with quaternary ammonium salt groups has better antibacterial properties than unmodified MMT [71], mainly at the early stages of bacterial growth [32]. Two stages in film degradation have been proposed: first, water diffusion and swelling occur, leading to microbial growth on the film; and, after that, enzymes and other compounds secreted by microorganisms result in weight loss and the breakdown of films. More pronounced degradation when water adsorption is high has been reported in starch-based nanocomposites. It has been attributed to the formation of strong hydrogen bonds between the hydroxyl groups of MMT and starch that increase matrix cohesiveness [50]. In chickpea flour films, 10% Cloisite 20A films showed significantly lower swelling values than the other films (Table 1), and this fact could also contribute to their higher stability for biodegradation. In addition, the most degraded samples (5% halloysite and 5% bentonite films) corresponded to those with high swelling values. However, 10% bentonite film presented high swelling, and its degradation was similar to that of the control sample. Other factors, probably related to nanoclay composition, might affect the biodegradation behavior of films.

## 4. Conclusions

The addition of nanoclays to chickpea flour film-forming solution was found to be a valuable method to improve the properties of the films, although the effects were affected by the type (halloysite, bentonite, or Cloisite 20A) and concentration of nanoclay. All films were highly biodegradable. Especially at the highest concentration, 10% (*w*/*w*) on a chickpea flour basis, films were less soluble, more opaque and rigid, and exhibited higher thermal stability than those without any addition for all the nanoclays. However, the type of nanofiller had a great influence on film characteristics. Both hydrophilic nanoclays, halloysite and bentonite, contributed to the more ordered structure of starch, according to the results of FTIR spectroscopy.

Bentonite showed a better distribution in film structure than the other nanoclays and, at the highest concentration, was the nanoclay that most modified the properties of chickpea flour films. It improved tensile and puncture strengths and reduced the elasticity and water vapor permeability of the films, probably due to the higher degrees of intercalation in the film matrix and interaction with starch, the main component of chickpea flour. The chickpea film with bentonite can be considered an environmentally friendly material to extend the shelf life of packaged foods, and it will be very useful to study their possible application in foods where it is important to control the loss of water, such as fruit and vegetables.

## Figures and Tables

**Table 1:** Water vapor permeability, thickness, dry matter, solubility, swelling, and density of films.

Film	WVP ^1^	Thickness μm	Dry Matter (g/100 g)	Solubility (% D.M. ^2^)	Swelling (%)	Density (%)
Control	0.036 ± 0.001 a	114.08 ± 1.33 b	78.88 ± 1.30	30.77 ± 1.10 a	98.55 ± 2.28 c	1.58 ± 0.02 b
H5 ^3^	0.032 ± 0.003 ab	119.03 ± 2.41 ab	79.68 ± 1.35	29.37 ± 0.63 b	102.04 ± 1.0 c	1.65 ± 0.01 a
H10 ^4^	0.031 ± 0.002 ab	118.68 ± 6.67 ab	80.37 ± 1.46	27.93 ± 0.49 cd	93.28 ± 0.83 d	1.65 ± 0.02 a
B5 ^5^	0.030 ± 0.003 b	117.76 ± 0.57 ab	80.15 ± 0.27	28.87 ± 0.74 bc	120.59 ± 0.27 a	1.56 ± 0.02 bc
B10 ^6^	0.021 ± 0.001 c	114.33 ± 0.50 b	80.13 ± 1.30	28.23 ± 0.58 bcd	107.51 ± 4.07 b	1.65 ± 0.04 a
C5 ^7^	0.033 ± 0.002 ab	123.42 ± 8.07 a	79.73 ± 1.12	28.77 ± 0.77 bc	91.42 ± 4.06 d	1.53 ± 0.03 c
C10 ^8^	0.034 ± 0.002 ab	124.67 ± 1.92 a	79.24 ± 0.75	27.49 ± 0.42 d	82.91 ± 3.86 e	1.56 ± 0.02 bc

^1^ WVP, water vapor permeability expressed in ng/Pa s m; ^2^ D.M., dry matter; ^3^ H5 films with 5% halloysite; ^4^ H10, films with 10% halloysite; ^5^ B5, films with 5% bentonite; ^6^ B10, films with 10% bentonite; ^7^ C5, films with 5% Cloisite 20A; ^8^ C10, films with 10% Cloisite 20A. Means in the same column with different letters (a–e) are significantly different (*p* < 0.05).

**Table 2:** CIE L*a*b* color parameters, total color difference (Δ*E*), yellowness index (*YI*), whiteness index (*WI*), and opacity of films.

Film	L*	a*	b*	Δ*E*	*YI*	*WI*	Opacity
Control	91.41 ± 0.21 bd	−1.66 ± 0.11 c	16.65 ± 0.72 b	0.00 ± 0.00 d	26.03 ± 1.19 b	81.19 ± 0.73 b	14.99 ± 0.29 c
H5 ^1^	92.12 ± 0.13 a	−1.70 ± 0.06 c	14.42 ± 0.40 c	2.18 ± 0.34 b	22.39 ± 0.69 c	83.43 ± 0.50 a	16.74 ± 0.10 b
H10 ^2^	91.53 ± 0.20 b	−1.73 ± 0.00 c	16.63 ± 0.91 b	2.00 ± 0.48 bc	25.99 ± 1.47 b	81.25 ± 0.89 b	17.65 ± 0.07 a
B5 ^3^	91.23 ± 0.14 bd	−1.33 ± 0.08 b	16.82 ± 0.71 b	1.40 ± 0.32 c	26.36 ± 1.13 b	80.98 ± 0.69 b	16.72 ± 0.31 b
B10 ^4^	90.77 ± 0.18 c	−1.02 ± 0.03 a	17.26 ± 0.29 ab	1.75 ± 0.28 bc	27.18 ± 0.50 ab	80.40 ± 0.34 bc	17.70 ± 0.10 a
C5 ^5^	91.13 ± 0.13 d	−1.62 ± 0.05 c	17.34 ± 0.53 ab	1.55 ± 0.20 c	27.21 ± 0.84 ab	80.45 ± 0.48 bc	17.07 ± 0.48 b
C10 ^6^	90.56 ± 0.24 c	−1.42 ± 0.05 b	18.24 ± 0.68 a	3.06 ± 0.58 a	28.79 ± 1.14 a	79.42 ± 0.71 c	17.53 ± 0.12 a

^1^ H5, films with 5% halloysite; ^2^ H10, films with 10% halloysite; ^3^ B5, films with 5% bentonite; ^4^ B10, films with 10% bentonite; ^5^ C5, films with 5% Cloisite 20A; ^6^ C10, films with 10% Cloisite 20A. Means in the same column with different letters (a–d) are significantly different (*p* < 0.05).

**Table 3:** Mechanical properties of chickpea flour films added to nanoclays.

Film	Tensile Strength at Maximum (MPa)	Elongation at Break (%)	Elastic Modulus (N/mm)	Puncture Strength (MPa)	Puncture Deformation (%)
Control	3.88 ± 0.12 e	26.75 ± 1.16 b	1.12 ± 0.03 d	4.14 ± 0.38 cd	9.53 ± 0.86 ab
H5 ^1^	4.24 ± 0.24 ce	32.07 ± 0.35 a	1.28 ± 0.10 d	4.52 ± 0.05 bc	10.01 ± 0.21 a
H10 ^2^	4.42 ± 0.16 c	31.02 ± 1.48 a	1.29 ± 0.17 d	4.19 ± 0.15 bcd	9.04 ± 0.26 bc
B5 ^3^	5.13 ± 0.21 b	25.40 ± 1.07 b	1.90 ± 0.03 b	4.58 ± 0.21 b	7.96 ± 0.62 de
B10 ^4^	6.26 ± 0.38 a	21.32 ± 1.02 c	2.85 ± 0.20 a	5.10 ± 0.14 a	6.14 ± 0.22 f
C5 ^5^	3.96 ± 0.17 de	25.46 ± 0.98 b	1.10 ± 0.07 d	3.53 ± 0.15 e	8.39 ± 0.32 cd
C10 ^6^	4.32 ± 0.19 cd	21.09 ± 0.58 c	1.58 ± 0.21 c	3.98 ± 0.34 d	7.54 ± 0.23 e

^1^ H5, films with 5% halloysite; ^2^ H10, films with 10% halloysite; ^3^ B5, films with 5% bentonite; ^4^ B10, films with 10% bentonite; ^5^ C5, films with 5% Cloisite 20A; ^6^ C10, films with 10% Cloisite 20A. Means in the same column with different letters (a–f) are significantly different (*p* < 0.05).

**Table 4:** DPPH radical scavenging capacity, FTIR ratios, and thermogravimetric analysis (TGA) results of films.

Film	DPPH (mg/g Film)	993/1020 cm^−1^ Ratio	1043/1020 cm^−1^ Ratio	TGA Third Stage Temperature Peak ^1^ (°C)	TGA Residual Weight (%)
Control	0.73 ± 0.02 be	1.77 ± 0.13 b	0.90 ± 0.05 a	287.03 ± 0.80 c	19.59 ± 0.84 d
H5 ^2^	0.65 ± 0.03 cd	1.63 ± 0.11 b	0.55 ± 0.07 d	288.84 ± 0.92 b	22.46 ± 0.30 b
H10 ^3^	0.59 ± 0.02 d	2.51 ± 0.12 a	0.76 ± 0.11 bc	289.19 ± 0.39 b	24.30 ± 0.15 a
B5 ^4^	0.92 ± 0.07 a	1.73 ± 0.24 b	0.69 ± 0.08 c	288.79 ± 0.51 b	21.85 ± 0.22 bc
B10 ^5^	0.67 ± 0.04 ce	2.31 ± 0.05 a	0.88 ± 0.03 ab	290.04 ± 0.24 ab	23.64 ± 0.97 a
C5 ^6^	0.78 ± 0.01 b	1.63 ± 0.27 b	0.79 ± 0.09 abc	288.95 ± 0.93 b	21.08 ± 0.38 c
C10 ^7^	0.76 ± 0.03 b	1.34 ± 0.12 c	0.74 ± 0.09 bc	290.81 ± 1.11 a	22.64 ± 0.54 b

^1^ The temperature peak is the value of the peak in the derivative thermogram obtained from the TGA curve. ^2^ H5, films with 5% halloysite; ^3^ H10, films with 10% halloysite; ^4^ B5, films with 5% bentonite; ^5^ B10, films with 10% bentonite; ^6^ C5, films with 5% Cloisite 20A; ^7^ C10, films with 10% Cloisite 20A. Means in the same column with different letters (a–e) are significantly different (*p* < 0.05).

## Data Availability

Data is contained within the article.

## Supplementary Materials

The following supporting information can be downloaded at: https://www.mdpi.com/article/10.3390/foods13010075/s1. Figure S1: Surface roughness measured from SEM images of chickpea flour films added to nanoclays.
