# Supplementary material for: Impact of Nanoclays Addition on Chickpea (Cicer arietinum L.) Flour Film Properties
Source: Foods. 2023 Dec 25;13(1):75. doi: 10.3390/foods13010075 (PMC10778780; doi:10.3390/foods13010075)
Supplement: Supplementary file 1 [file foods-13-00075-s001.zip › foods-2784625-supplementary.pdf]

Supplementary materials. Figure S1

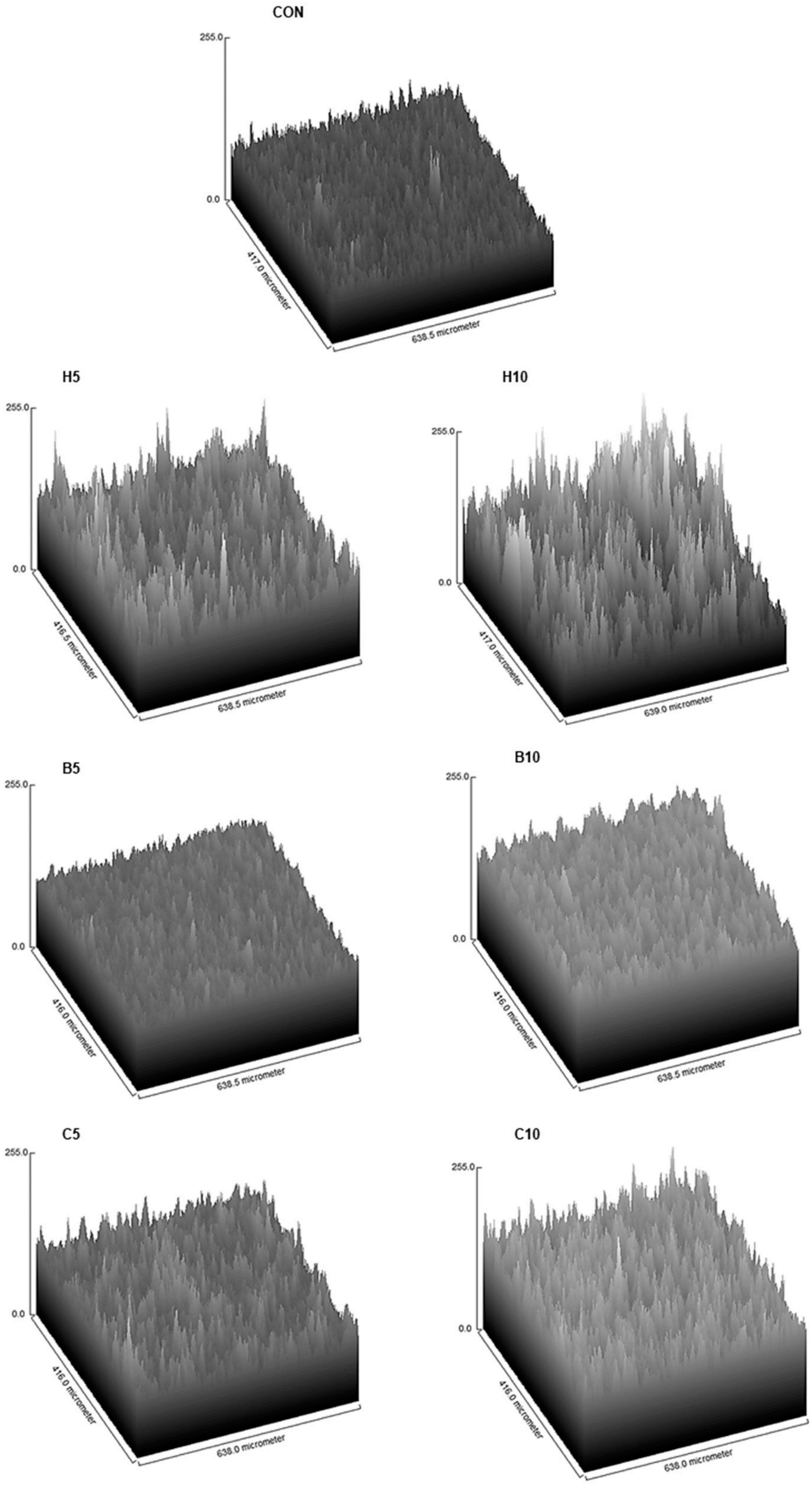

Figure S1. Surface roughness measured from SEM images of chickpea flour films added of nanoclays. CON, control film; H5, film with 5% halloysite; H10, film with 10% halloysite; B5, film with 5% bentonite; B10, film with 10% bentonite; C5, film with 5% Cloisite 20A; C10, film with 10% Cloisite 20A.
